# Recent Advances of Graphitic Carbon Nitride-Based Structures and Applications in Catalyst, Sensing, Imaging, and LEDs

**DOI:** 10.1007/s40820-017-0148-2

**Published:** 2017-06-08

**Authors:** Aiwu Wang, Chundong Wang, Li Fu, Winnie Wong-Ng, Yucheng Lan

**Affiliations:** 1 School of Optical and Electronic Information, Huazhong University of Science and Technology, Wuhan, 430074 People’s Republic of China; 2 Center of Super-Diamond and Advanced Films (COSDAF) and Department of Physics and Materials Science, City University of Hong Kong, 83 Tat Chee Avenue, Kowloon, Hong Kong SAR People’s Republic of China; 3 College of Materials and Environmental Engineering, Hangzhou Dianzi Univerisity, Hangzhou, 310018 People’s Republic of China; 4 Materials Science Measurement Division, National Institute of Standards and Technology, Gaitherburg, MD 20899 USA; 5 Department of Physics and Engineering, Morgan State University, Baltimore, MD 21251 USA

**Keywords:** Graphitic carbon nitride (g-C_3_N_4_), Catalysis, Sensing, Imaging, LED

## Abstract

The graphitic carbon nitride (g-C_3_N_4_) which is a two-dimensional conjugated polymer has drawn broad interdisciplinary attention as a low-cost, metal-free, and visible-light-responsive photocatalyst in the area of environmental remediation. The g-C_3_N_4_-based materials have excellent electronic band structures, electron-rich properties, basic surface functionalities, high physicochemical stabilities and are “earth-abundant.” This review summarizes the latest progress related to the design and construction of g-C_3_N_4_-based materials and their applications including catalysis, sensing, imaging, and white-light-emitting diodes. An outlook on possible further developments in g-C_3_N_4_-based research for emerging properties and applications is also included.

## Highlights

- The g-C3N4-based materials have excellent electronic band structures, electron-rich properties, basic surface functionalities, high physicochemical stabilities and are “earth-abundant.”
- Recent progress of g-C3N4-based nanostructures in catalyst, sensing, imaging and LEDs have been discussed in details.
- An outlook on possible further developments in g-C3N4-based research for emerging properties and applications is also included.

## Introduction

Graphitic carbon nitride (g-C_3_N_4_), one of the oldest reported polymers in the literature, has a general formula of (C_3_N_3_H)_n_. The history of development could be traced back to 1834 [1]. Research work has been inspired in the 1990s due to a theoretical prediction that diamond-like β-C_3_N_4_ could have extremely high hardness values [2]. At ambient conditions, g-C_3_N_4_ is regarded as the most stable allotrope. Similar to graphite, g-C_3_N_4_ is a layered material in which van der Waals force holds the stacking layers (covalent C–N bonds) and each layer is composed of tri-s-triazine units connected with planar amino groups [3]. The tri-s-triazine ring structure provides the polymer a high thermal stability (600 °C in air) and chemical stability in both acidic and alkaline environments [4].

Utilization of g-C_3_N_4_ in the heterogeneous catalysis arena started around a decade ago [5, 6]. The discovery of g-C_3_N_4_ polymer as a metal-free conjugated semiconductor photocatalysis for water splitting was first reported by Wang et al. [7] due to its appealing electronic structure, i.e., having a modulated bandgap and being an indirect semiconductor. Since then, these unique properties of g-C_3_N_4_ make it a promising candidate for visible-light photocatalytic applications utilizing solar energy. Solar energy is attracting worldwide attention by providing about 120,000 TW annually to the earth as one of the green, clean, and sustainable energy resources. Solar-induced chemical processes would be able to greatly extend the applications of g-C_3_N_4_. Since the landmark discovery of photocatalytic water splitting using TiO_2_ electrodes by Fujishima in 1972, photocatalytic technology has been regarded as one of the most important strategies to address global energy and environmental issues [8]. Since then, there have been numerous developments in the fabrication of highly efficient semiconductor-based photocatalysts such as metal-based oxides and sulfides [9–12].

Notably, g-C_3_N_4_ has become a new family of next generation, non-toxic, metal-free, earth-abundant, and visible-light-driven polymeric semiconductor for applications in the degradation of organic pollutants, hydrogen evolution from water, sensing, imaging, and energy conversion [5, 9, 13–144]. Many reviews can be found mainly focusing on synthesis and catalytic applications of g-C_3_N_4_ [39–45, 145, 146]. However, a systematic description of the catalyst (photo and organic), bio-imaging, (chemical and bio-) sensing, devices, and energy-related applications (batteries, supercapacitors, white-light-emitting diodes, and oxygen reduction reaction) of g-C_3_N_4_ has not been presented until now. In this review, we give an overview of the porosity, luminescence, conductivity, and catalytic properties of g-C_3_N_4_, as well as their bio-imaging, photodynamic therapy, chemical sensing, and white-light-emitting diode applications. We believe this is the dawn for the development of g-C_3_N_4_. There are still new physical properties yet to be discovered based on g-C_3_N_4_ nanostructures. We are at a critical time to highlight the progress and provide a good source of references for this booming research topic.

## g-C_3_N_4_-Based Structures

**Fig. 1:**
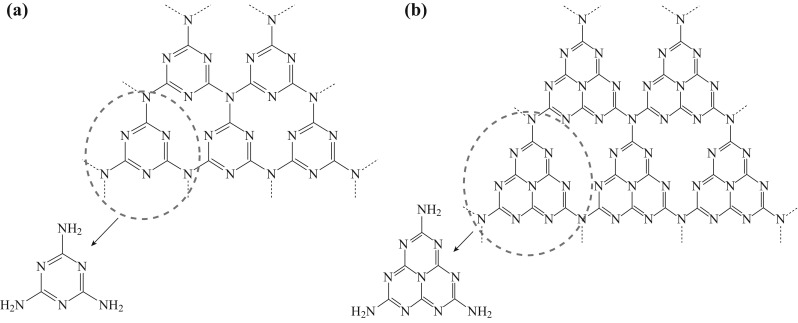
**a** Triazine and **b** tri-s-triazine (heptazine) structures of g-C_3_N_4_. Reprinted with permission from Ref. [40]. Copyright 2016 American Chemical Society

First-principle calculations predicted seven phases of g-C_3_N_4_, namely α-C_3_N_4_ (bandgap of 5.5 eV), β-C_3_N_4_ (bandgap of 4.85 eV), cubic C_3_N_4_ (bandgap of 4.3 eV), pseudocubic C_3_N_4_ (bandgap of 4.13 eV), g-h-triazine (bandgap of 2.97 eV), g-o-triazine (bandgap of 0.93 eV), and g-h-heptazine (bandgap of 2.88 eV) [4]. Figure 1 shows the primary tectonic units, triazine and tri-s-triazine ring structures, for forming the allotropes of g-C_3_N_4_. The structure can be viewed as graphite whose carbon lattice is partially substituted with nitrogen atoms in a regular fashion. g-C_3_N_4_ is an *n*-type semiconductor [5–7], which intrinsically possesses a very high nitrogen content dominated by a pyridinic and graphitic nitrogen while supported by a two-dimensional (2D) graphene sheet or three-dimensional (3D) porous graphitic carbon. The structure of g-C_3_N_4_ can be controlled by a variety of synthetic routes, including different condensation temperature, different ratio of precursors, porosity induced by hard/soft templating strategies, and exfoliation and doping. Synthetic routes and various morphologies including bulk, mesoporous, 3D, 2D, one-dimensional (1D), and zero-dimensional (0D) g-C_3_N_4_ will be discussed in the following.

### Synthetic Routes of g-C_3_N_4_

g-C_3_N_4_ can be synthesized by thermal polymerization of abundant nitrogen-rich and oxygen-free compound precursors containing pre-bonded C–N core structures (triazine and heptazine derivatives) such as urea [46], melamine [47–49], dicyandiamide [50–54], cyanamide [14, 35, 55, 56], thiourea [57, 58], guanidinium chloride [59–61, 125], guanidine thiocyanate [126], and thiourea oxide [127]. The condensation pathways from above C/N precursors are facile and efficient routes to form the polymeric g-C_3_N_4_ network [39]. Many reports discussed that different types of precursors and treatments can strongly influence the physicochemical properties of the resulting g-C_3_N_4_, including surface area, porosity, absorption, photoluminescence, C/N ratio, and nanostructures.

**Fig. 2:**
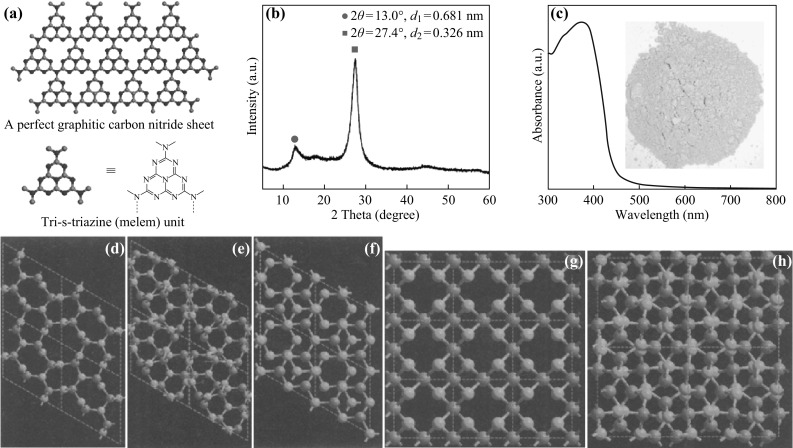
Crystal structure and optical properties of graphitic carbon nitride. **a** Schematic diagram of a perfect graphitic carbon nitride sheet constructed from melem units. **b** Experimental XRD pattern of the polymeric carbon nitride, revealing a graphitic structure with an interplanar stacking distance of aromatic units of 0.326 nm. **c** UV-visible diffuse reflectance spectrum and image (*inset*) of g-C_3_N_4_. Reprinted with permission from Ref. [5]. Copyright 2009 Nature Publishing Group. Representation of the β-C_3_N_4_ (**d**), α-C_3_N_4_ (**e**), graphite-C_3_N_4_ (**f**), pseudocubic-C_3_N_4_ (**g**), and cubic-C_3_N_4_ (**h**). The carbon and nitrogen atoms are depicted as *gray* and *blue spheres*, respectively, from Ref. [4]. Copyright 1996 American Association for the Advancement of Science. (Color figure online)

 Various surface modifications and functionalities have been employed to obtain desired structures such as 3D bulks, 2D nanosheets, 2D films, 1D nanorods, 1D nanotubes, 1D nanowires, and 0D quantum dots. For example, the urea precursor can be transformed to g-C_3_N_4_ at ca. 550 °C, as confirmed by X-ray diffraction (XRD, Fig. 2a, b). The C_3_N_4_ powders are usually yellow under the visible light. The optical properties are shown in Fig. 2c. The polymeric g-C_3_N_4_ is unstable at above 600 °C. Beyond 700 °C, g-C_3_N_4_ produces nitrogen and cyano fragments.

General analytical techniques for confirming the presence of g-C_3_N_4_ include X-ray photoelectron spectroscopy (XPS), XRD, and Fourier transform infrared (FTIR) spectroscopy [40]. The density function theory (DFT) calculations are used to reveal the characteristics of the valence and conduction bands. For example, g-C_3_N_4_ is found to mainly composed of the nitrogen *p*_Z_ orbitals and carbon *p*_Z_ orbitals, respectively [5].

### Porosity of g-C_3_N_4_

High surface area and continuous porosity (as active centers) are important requirements for catalysis, gas, and energy storage technologies. Introduction of porosity in g-C_3_N_4_ can significantly increase their exposed surface area and accessible channels, and active edges, thus promoting the charge separation, molecular mass transfer, light harvesting, and surface reactions [145]. All these advantageous features can benefit the enhancement of photocatalytic efficiency. It is well known that the porous carbons have outstanding properties with respect to their use in energy applications, including as electrode materials for supercapacitors and as materials for solid-state hydrogen and carbon dioxide storage. The attractive attributions of porous carbons include low cost, environmental friendliness, chemical and thermal stability, easiness of processing, and low framework density. The activated carbons have been traditionally employed as absorbents or catalyst supports [62]. Compared with activated carbons, g-C_3_N_4_ has a moderate nitrogen content and ideal stoichiometry. The nitrogen content induces their unique surface properties such as semiconducting character, mechanical stability, thermal and chemical stability, which are superior to all other carbon nanomaterials (Table 1).

**Table 1:** Recent reports on g-C_3_N_4_-based photocatalysts

Catalysts composition	Precursors of g-C_3_N_4_	Photocatalyst applications	Ref. (year)
g-C_3_N_4_	Cyanamide	Hydrogen production	[5] (2009)
g-C_3_N_4_/Graphene/NiFe	Urea	Photoelectrochemical	[13] (2016)
g-C_3_N_4_ nanocapsules	Cyanamide	Hydrogen production	[14] (2017)
g-C_3_N_4_/Co–N	Urea	Hydrogen production	[16] (2016)
g-C_3_N_4_/Graphene	Dicyandiamide	Hydrogen production	[18] (2014)
g-C_3_N_4_/PDA	Melamine	Hydrogen production	[20] (2015)
Alkalinized-C_3_N_4_/Fe	Melamine	Photo degradation	[21] (2016)
g-C_3_N_4_/Fe_3_O_4_	Melamine	Photo degradation	[22] (2013)
g-C_3_N_4_ Film	Melamine	Photoelectrochemical	[24] (2015)
g-C_3_N_4_/AgBr	Melamine	Photo degradation	[25] (2015)
g-C_3_N_4_ nanofibers	Melamine	Photo degradation	[30] (2013)
g-C_3_N_4_/PNA	Melamine	Photo degradation	[31] (2013)
g-C_3_N_4_ Film	Cyanamide	Photoelectrochemical	[32] (2015)
P-doped g-C_3_N_4_	Melamine	Hydrogen production	[33] (2015)
Amorphous g-C_3_N_4_	Dicyandiamide	Hydrogen production	[34] (2015)
g-C_3_N_4_	Cyanamide	Hydrogen Peroxide production	[35] (2014)
g-C_3_N_4_/Ag/TiO_2_	Melamine	Photo degradation	[36] (2014)
g-C_3_N_4_/Bi	Urea	NO Purification	[37] (2015)
g-C_3_N_4_/TiO_2_	Melamine	Photoelectrochemical	[47] (2016)
g-C_3_N_4_/ZIF	Melamine	CO_2_ Reduction	[48] (2015)
N-doped g-C_3_N_4_	Melamine	Hydrogen production	[49] (2015)
Iodine modified g-C_3_N_4_	Dicyandiamide	Hydrogen production	[50] (2014)
Holey g-C_3_N_4_	Dicyandiamide	Hydrogen production	[51] (2015)
Phosphorylation g-C_3_N_4_	Dicyandiamide	Hydrogen production	[53] (2015)
Porous g-C_3_N_4_	Dicyandiamide	Photo degradation	[54] (2015)
g-C_3_N_4_	Cyanamide	NO decomposition	[55] (2010)
Mesoporous g-C_3_N_4_	Cyanamide	Hydrogen peroxide production	[56] (2015)
Porous g-C_3_N_4_	Thiourea	Photo degradation	[57] (2016)
S-doped g-C_3_N_4_	Thiourea and Melamine	CO_2_ reduction	[58] (2015)
g-C_3_N_4_/bismuth-based oxide	Melamine or guanidine hydrochloride	Photo degradation	[61] (2016)
g-C_3_N_4_/Graphene	Urea	Hydrocarbon oxidation	[66] (2016)
3D porous g-C_3_N_4_	Melamine	Photo degradation	[67] (2016)
g-C_3_N_4_ nanoplatelets	Melamine	Water splitting	[73] (2015)
Graphene-like g-C_3_N_4_ nanosheets	Dicyandiamide	Hydrogen production	[75] (2012)
Crystalline g-C_3_N_4_	Dicyandiamide	Hydrogen production	[76] (2014)
Sulfur-mediated g-C_3_N_4_	Trithiocyanuric acid	Water oxidation	[77] (2011)
Nanotube g-C_3_N_4_	Melamine	Photo degradation	[79] (2014)
Helical g-C_3_N_4_	Cyanamide	Hydrogen production	[80] (2014)
Nanorod g-C_3_N_4_	Cyanamide	Hydrogen production and photoenzymatic catalysis	[81] (2014)
Mesoporous g-C_3_N_4_ nanorods	Cyanamide	Hydrogen production and reduction of nitrophenol	[82] (2012)
PAN/g-C_3_N_4_	Melamine	Hydrogen production	[83] (2016)
g-C_3_N_4_/ZIF	Urea	Photo degradation	[84] (2017)
g-C_3_N_4_	Dicyandiamide	Photo degradation	[91] (2014)
g-C_3_N_4_/Pd	Cyanamide	Organic catalyst	[92] (2015)
Oxidized g-C_3_N_4_	Melamine	Organic synthesis	[95] (2016)
g-C_3_N_4_/GO	Melamine	Photo degradation	[98] (2014)

Cavities can be introduced to form mesoporous g-C_3_N_4_ frameworks [29]. Qiao’s group reported the preparation of flexible films by integrating 2D mesoporous g-C_3_N_4_ (SiO_2_ template method) with graphene sheets. A commonly used mesoporous silica, SBA15 was employed as a template to fabricate mesoporous g-C_3_N_4_ [6, 56]. However, hydrogen fluoride or other acid that are usually used to remove the host matrices of SiO_2_ is hazardous. Besides hard template methods [128–130], soft templates [131, 132] and bubble templates [133–136] are also utilized to fabricate porous g-C_3_N_4_. Liu et al. reported the preparation of porous g-C_3_N_4_ by a simple co-pyrolyzation of co-precursors melamine and NH_4_HCO_3_. NH_4_HCO_3_ not only enhanced the specific area by bubbles, but it also shifted the conduction band and promoted the separation of charge carriers [63]. Calcium salts have also been utilized as a template for the synthesis of porous g-C_3_N_4_ with enhanced surface properties [143]. Metal–organic framework (MOF) is a typical high surface area materials and has also been utilized as templates for porous g-C_3_N_4_. Pandiaraj et al. [64] have reported MOF-derived g-C_3_N_4_ porous nanostructures.

### Bulky g-C_3_N_4_

 Bulky g-C_3_N_4_ can be synthesized by thermal condensation of a variety of precursors such as cyanamide, dicyandiamide, melamine, thiourea or urea between 400 and 600 °C. For example, g-C_3_N_4_ was synthesized from cyanamide into a combination of addition and polycondensation, in which case the cyanamide molecules were condensed to dicyandiamide and melamine at 203 and 234 °C, respectively. Next, the condensed dicyandiamide was removed. Essentially, all melamine-based products were found when the temperature was about 335 °C. Further heating to about 390 °C resulted in the rearrangement of tri-mesotriazine units via melamine. Finally, the polymer g-C_3_N_4_ occurred at about 520 °C by further condensation of the unit, which is thermally unstable at temperatures above 600 °C. During the calcination, the color changed from white (precursor) to light yellow (500 °C) and then dark orange (650 °C) [5, 40]. However, g-C_3_N_4_ obtained by such methods usually possesses a relatively low surface area (10 m^2^ g^−1^) and poor water solubility. Furthermore, the bulk g-C_3_N_4_ does not exhibit any photoluminescence characteristics when dissolved in solvents.

It has recently been found that urea is an excellent precursor for the synthesis of flaky g-C_3_N_4_ having a high specific surface area and a high porosity. g-C_3_N_4_ was synthesized at various calcination temperatures between 450 and 600 °C in a muffle furnace for 2 h at a heating rate of 15 °C min^−1^ from oxygen-containing urea. During the thermal treatment process, the generated gases such as NH_3_ at a low temperature (<200 °C) and CO_2_ at a high temperature play a leading role in processing highly porous g-C_3_N_4_. The advantage of this method includes simplicity, convenience, and the absence of introduction of impurities during the synthesis of nanostructures. Compared to urea-derived g-C_3_N_4_, comprising of wrinkled porous 2D nanosheets, both thiourea-derived and dicyandiamide-derived g-C_3_N_4_ samples showed large sheets without porous structure. The specific surface area and crystallinity of g-C_3_N_4_ were marginally improved with increasing calcination temperatures. Generally, the heating rate is slower; the porosity of g-C_3_N_4_ could be improved [40].

Exfoliation methods such as sonication have been employed as a typical top-down route to obtain ultrathin g-C_3_N_4_ with excellent photoluminescence properties [65]. Bulk materials can be swelled and then exfoliated in the pure water. The dispersed ultrathin g-C_3_N_4_ nanosheets were negatively charged (zetapotential of about ~30.3 mV).

### Three-Dimensional g-C_3_N_4_-Based Micro/Nanostructures

**Fig. 3:**
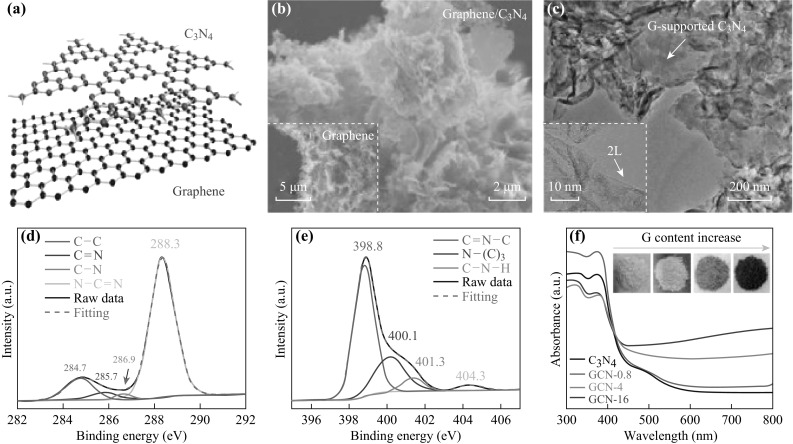
**a** Schematic 3D g-C_3_N_4_/Graphene structures. **b, c** SEM images of g-C_3_N_4_/graphene nanocomposites. *Inset* TEM image. **d** C 1*s*, **e** N 1*s* XPS and **f** UV–vis spectra of g-C_3_N_4_/graphene nanocomposites. *Insets*: photographs of the powder samples. Reprinted with permission from Ref. [66]. Copyright 2016 American Chemical Society

Three-dimensional architectures fabricated using nano-scale building blocks (0D, 1D, and 2D) are hot topics due to the desirable combination of high internal reactive surface area and straightforward molecular transport. However, the fabrication of 3D porous g-C_3_N_4_ has been a big challenge up to now. Recently, Liu’s group developed an efficient chemical vapor deposition growth strategy for 3D g-C_3_N_4_/graphene nanocomposites [66]. They found that g-C_3_N_4_ can be grown along the surface of graphene (see Fig. 3a, b, c). The C–C bonds (284.7 eV) and N–C=N bonds (288.3 eV) can be detected from the high-resolution C 1 s spectrum (Fig. 3d). Furthermore, sp^2^ aromatic C=N–C (398.8 eV), tertiary N-(C)_3_ (400.1 eV), and amino group C–N–H (401.3 eV) can be fitted in the high-resolution N 1 s spectrum (Fig. 3e). UV–vis spectroscopy (Fig. 3f) reveals the optical properties of the 3D composites to be similar to those of the pure g-C_3_N_4_.

Yuan et al. [67] reported the 3D porous g-C_3_N_4_ network assembled by exfoliated ultrathin nanosheets interconnected in large quantity via H_2_SO_4_ intercalation and subsequent thermal treatment.

### Two-Dimensional g-C_3_N_4_ Nanostructures

**Fig. 4:**
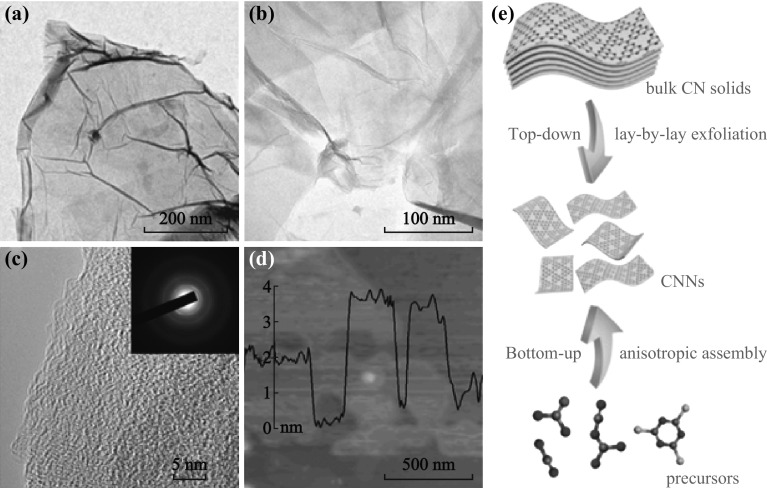
**a-c** TEM images and **d** AFM image of g-C_3_N_4_ nanosheets. *Inset* of **c** SAED pattern. Reprinted with permission from Ref. [52]. Copyright 2014 John Wiley and Sons. **e** Schematic illustration of top-down and bottom-up synthetic strategies for g-C_3_N_4_ nanosheets. Reprinted with permission from Ref. [68]. Copyright 2015 The Royal Society of Chemistry. (Color figure online)

Two-dimensional materials have received tremendous attention in the past decade because their ultimate structure was reported by Geim [69]. The topic about graphene has been cited over 30,000 times up to now (Google Scholar), and graphene has been employed widely in energy applications (such as lithium-ion batteries and supercapacitors [70–72]). Similar to graphene, g-C_3_N_4_ also has a typical sp^2^ network (graphite-like layer structure) with weak van der Waals interactions across the layers. Inspired by the successful exfoliation of graphene from bulk graphite, Xie et al. [65] firstly demonstrate that ultrathin g-C_3_N_4_ nanosheets could be prepared by a green liquid exfoliation from bulk g-C_3_N_4_ in water. From bulk to ultrathin nanosheets (several layers), g-C_3_N_4_ nanosheets show an obvious increase in density of states (DOS) at the conduction band edge with respect to the bulk counterpart by first-principle density-functional calculations. Global effects have been launched to synthesize single or few-layer g-C_3_N_4_ nanosheets due to their attractive physicochemical properties [52, 73, 74]. Figure 4 shows characterization of the g-C_3_N_4_ nanosheets studied by transmission electron microscopy (TEM) and atomic force microscopy (AFM), illustrating the similarity of ultrathin layers of exfoliated g-C_3_N_4_ to that of graphene.

There are two other synthetic approaches of 2D g-C_3_N_4_ nanosheets: one is known as the top-down approach, and the other one is the bottom-up approach (Fig. 4e). For the top-down approach, chemical etching and ultrasonication-assisted liquid exfoliation are the two main technologies involved [75, 76]. Template-assisted method and heteroatom-mediated method have been commonly employed for the bottom-up approaches [77, 78]. The big atomic size of sulfur can influence the conformation and the connectivity of the resultant g-C_3_N_4_ and hence offer a template tool to tune the texture and electronic structure. The first report of template-mediate synthesis of 2D g-C_3_N_4_ nanosheets involved the use of GO-derived silica [78]. Zhang et al. [23] reported the sol processing for the fabrication of g-C_3_N_4_ thin films with HNO_3_ as a strong oxidizing acid. Liu et al. [54] developed a method to grow g-C_3_N_4_ thin films directly on conductive substrates.

### One-Dimensional g-C_3_N_4_ Nanostructure (Nanorods, Nanotube, and Nanofibers)

**Fig. 5:**
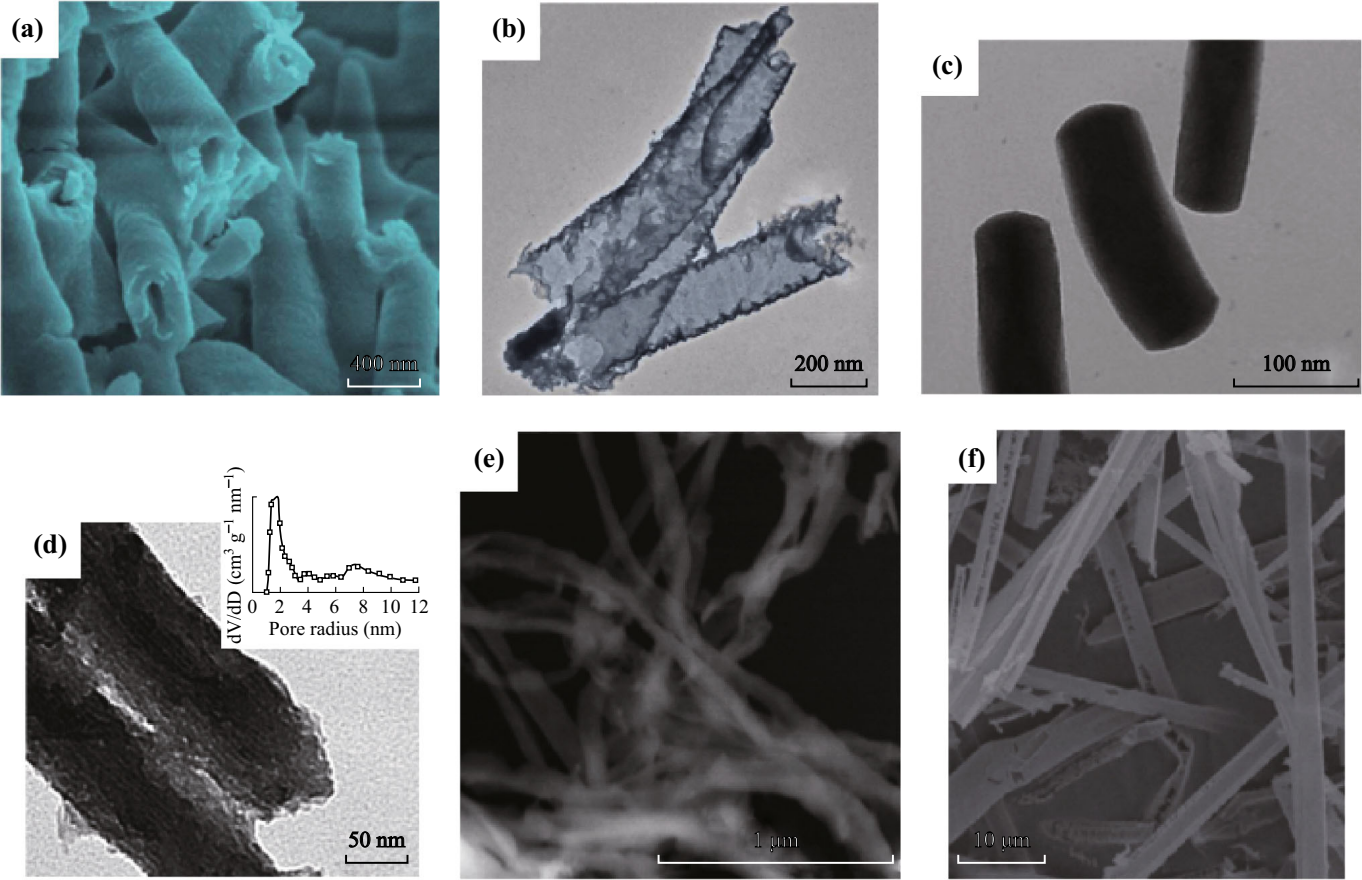
1D g-C_3_N_4_ nanostructures. **a** SEM image of nanotubes [79]. **b** TEM image of nanotubes [83]. **c** TEM image of nanorods [81]. **d** TEM image of porous nanorods [82]. Inset: pore size distribution. **e** SEM image of nanofibers [30]. **f** SEM image of tubular structures [85]. Reprinted with permission from Ref. [79] (Copyright 2014 The Royal Society of Chemistry), with permission from Ref. [83] (Copyright 2016 American Chemical Society), with permission from Ref. [81] (Copyright 2014 American Chemical Society), with permission from Ref. [82] (Copyright 2012 The Royal Society of Chemistry), with permission from Ref. [30] (Copyright 2013 American Chemical Society), with permission from Ref. [85] (Copyright 2016 Wiley–VCH Verlag GmbH), respectively

One-dimensional g-C_3_N_4_ nanostructures hold good promise for electronic and electrochemical performances due to their high surface area, and light harvesting and mass transfer properties. Wang et al. described a facile method to fabricate g-C_3_N_4_ nanotubes (Fig. 5a) by directly heating melamine without any templates. The resulting nanotubes exhibited a blue fluorescence and excellent photocatalytic properties [79]. Hollow 1D g-C_3_N_4_ nanostructures have been developed using a sulfur-mediated self-templating method by Liu’s group [83], as shown in Fig. 5b.

Liu et al. [81] also reported the synthesis of g-C_3_N_4_ nanorods by using chiral silica nanorods as templates for practical enzymatic applications. In addition, Li et al. [82] described a one-step method to fabricate mesoporous g-C_3_N_4_ nanorods with the template SBA-15. Tahir et al. [30] reported the synthesis of g-C_3_N_4_ nanofibers for energy storage and Photo degradation applications. Recently, Tong et al. combined the hydrothermal and condensation techniques to obtain a tubular g-C_3_N_4_ isotype heterojunction with excellent photocatalytic property [85]. Figure 5c–f depicts these tubular nanostructures.

**Fig. 6:**
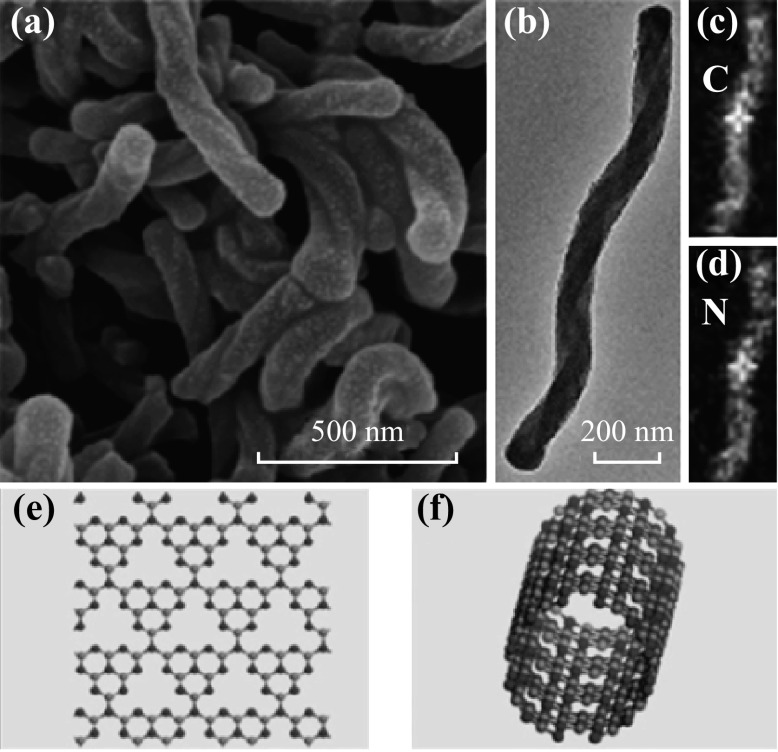
Morphology characterization of the HR-CN sample. **a** SEM, **b** TEM, and **c**, **d** corresponding elemental mapping images of g-C_3_N_4_. Reproduced from Ref. [80] by permission of the John Wiley & Sons Ltd. **e** A g-C_3_N_4_ layer and **f** A single g-C_3_N_4_ nanotube formed by rolling the g-C_3_N_4_ layer. Reproduced from Ref. [79] by permission of the Royal Society of Chemistry

Zheng et al. [80] developed a nanocasting technique to fabricate twisted hexagonal rod-like C_3_N_4_ by using chiral silicon dioxide as templates. The helix is an important template in nature, as one finds it in DNA, RNA, and proteins. Synthesis of chiral inorganic nanostructures has recently gained considerable attention. The chiral nanostructures shown in Fig. 6a–f reveal the helical morphology and ordered channels winding around the rod centers. This is the first report about both left- and right-handed chiral nanostructures with unique optical activities.

### Zero-Dimensional g-C_3_N_4_ Nanostructure

**Fig. 7:**
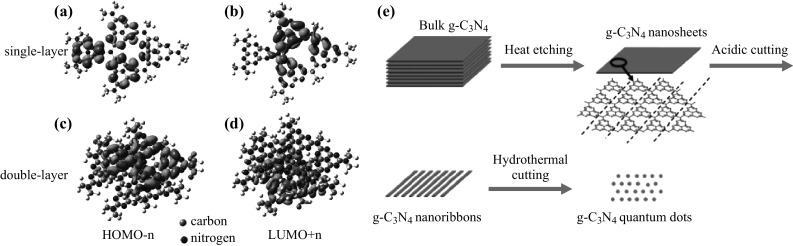
**a** HOMO-n and **b** LUMO + n orbitals of the single-layered g-C_3_N_4_, respectively. **c** HOMO-n and **d** LUMO + n orbitals of the double-layered g-C_3_N_4_, respectively. Reproduced from Ref. [87] by permission of John Wiley & Sons Ltd. **e** Schematic illustration of the controllable synthesis of g-C_3_N_4_ nanosheets, nanoribbons, and quantum dots. Reproduced from Ref. [86] by permission of the Royal Society of Chemistry

When the size of the nanostructure is less than 10 nm, g-C_3_N_4_ nanostructures (typically contain a few thousand atoms) usually show significant quantum confinement effects and possess excellent properties like bright fluorescence, water solubility, and above all, non-toxicity. This is in contrast to the fact that most essential elements in semiconductor quantum dots (QDs) (for example, Se in CdSe, Pb in PbTe, and Te in CdTe) all have risks of long-term toxicity and potential environmental hazards. Similar to the synthetic routes of 2D g-C_3_N_4_ nanosheets, g-C_3_N_4_ QDs have been mainly synthesized by top-down and bottom-up approaches.

g-C_3_N_4_ QDs were prepared first by the top-down approach. Wang et al. was the first to prepare g-C_3_N_4_ QDs by a thermal-chemical etching process from bulk g-C_3_N_4_, as illustrated in Fig. 7e [86]. Xie et al. demonstrated an exfoliation strategy for the preparation of single-layered QDs. When these QDs passed through the cell membranes, they exhibited an excellent two-photon absorption behavior as compared with the double-layered QDs [87] (Fig. 7a–d). Recently, Wu et al. developed a doping method (phosphorus as dopant) to control the emission wavelength of g-C_3_N_4_ QDs to be in the whole visible-light regime [88]. This was the first report that phosphorus doping could change the direct bandgap of g-C_3_N_4_ QDs.

Hydrothermal and microwave heating methods are commonly utilized for the synthesis of g-C_3_N_4_ QDs as the bottom-up approach [89, 90]. Lu et al. [90] reported the synthesis of g-C_3_N_4_ QDs with strong blue photoluminescence by hydrothermal heating of the citric acid and thiourea. Cao et al. [89] developed a facile microwave-assisted fabrication of g-C_3_N_4_ QDs. Compared with the majority of carbon materials such as graphene QDs and carbon QDs, g-C_3_N_4_ QDs which possess both nitrogen-rich and electron-rich properties and basic surface functionalities represent a new family of luminescent QDs.

## Multifunctional Applications

As the most stable allotrope of carbon nitride, g-C_3_N_4_ is versatile materials with unique semiconducting, excellent photocatalytic properties. They are also environmental friendly, low cost, and metal-free, which make them attractive for a range of applications beyond catalysis, including sensing, bio-imaging, photodynamic therapy, and energy conversion.

### g-C_3_N_4_ Catalysts

Polymeric g-C_3_N_4_ semiconductors are widely used as catalysts due to their excellent chemical stability and unique electronic band structure. In the formation of the g-C_3_N_4_ network, the C-*p*_z_ orbit composes the lowest unoccupied molecular orbital (LUMO), and N-*p*_z_ orbital composes the highest occupied molecular orbital (HOMO), with a 2.7 eV bandgap between these two orbitals [91]. This suitable bandgap can absorb the solar electromagnetic energy with wavelength less than 475 nm. It was found that pyrrolic nitrogen has the strongest role in acetylene hydrochlorination among all nitrogen species [17].

**Fig. 8:**
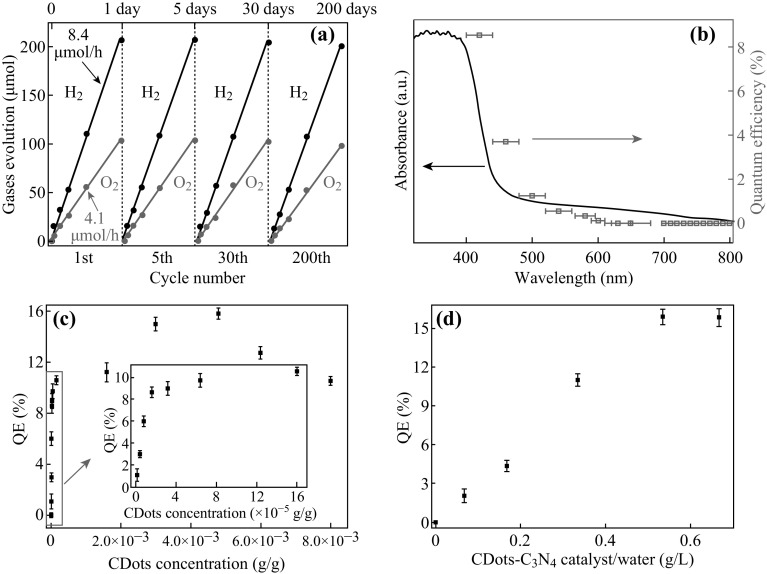
**a** Typical H_2_ and O_2_ production from water under visible-light irradiation. **b** Wavelength-dependent QE (*red dots*) of water splitting by composite catalyst. **c** QE for different concentrations of carbon dots/g-C_3_N_4_ catalysts in a fixed mass of composite catalyst. **d** QE for different catalyst loads with a constant carbon dot concentration in 150 ml of ultra-pure water. Reproduced with permission from Ref. [95]. Copyright 2015 American Association for the Advancement of Science. (Color figure online)

The rapid recombination rate of electron–hole pairs results in low efficiency, thus limiting the practical applications of g-C_3_N_4_. Sun et al. [92] developed a homogeneous catalyst, Pd/g-C_3_N_4_, for a Suzuki–Miyaura coupling reaction with superior catalytic activity under mild conditions. It is well known that the Suzuki–Miyaura coupling reaction is of primary importance for the construction of C–C bonds. The uniform Pt nanoparticles deposited on the surface of g-C_3_N_4_ networks can result in a high yield of 97% biphenyl and 100% bromobenzene. Kumar et al. reported a nanocomposite of an iron(II) bipyridine with carbon nitride as a photocatalyst for the oxidative coupling of benzylamines under mild reaction conditions, resulting in excellent activity and effective recycling ability [93]. A photoactive catalyst Ru/g-C_3_N_4_ was developed by Sharma et al. [94] for efficient photocatalytic transfer hydrogenation of aldehydes and ketones under mild conditions. Xie’s group explored the generation of singlet oxygen in oxidized g-C_3_N_4_ [95].

g-C_3_N_4_ can be used as a new kind of metal-free photocatalysts. Wang et al. [5] was among the first to use g-C_3_N_4_ as a photocatalyst for hydrogen production from water. However, the quantum efficiency of the catalyst is only 0.1% with the irradiation of 420–460 nm due to its fast recombination of electron–hole pairs. To solve this problem, modified 2D g-C_3_N_4_ materials with a redshift absorption were produced and a quantum efficiency of 8.8% was achieved at 420 nm by the same group [96]. Liu et al. [97] developed a carbon dots/g-C_3_N_4_ nanocomposite as a metal-free photocatalyst with high quantum yield and excellent stability. The overall evolution of H_2_ and O_2_ is shown in Fig. 8a with a molar ratio of 2.02 (a value of 2 is identified for water splitting). Absorbance and quantum efficiency (QE) of the carbon dots/g-C_3_N_4_ nanocomposite were measured and are shown in Fig. 8b. The catalyst composition was optimized by measuring QE for different concentrations of carbon dots in a fixed mass of composite, as shown in Fig. 8c. With the increase in carbon dots, the QE can reach as high as 16% (Fig. 8d).

Recently, nanohybrids (van der Waals heterostructures) which compose of different 2D nanolayers exhibit much improved catalytic activities. For example, porous g-C_3_N_4_/graphene films have been fabricated as electrodes for efficient hydrogen evolution by Qiao et al. [29]. Dai et al. [98] obtained graphene oxide/g-C_3_N_4_ nanosheets by a sonication method with reinforced photocurrent. Ma et al. demonstrated the fabrication of hybrid g-C_3_N_4_/graphene quantum dot nanocomposites. The hybrid nanocomposites have an excellent efficiency for water splitting due to decreased bandgap. Experimental results and DFT calculations revealed that the chemical bonding of two different layered materials can improve the catalytic activity [18]. Xu et al. [137] fabricated AgBr/g-C_3_N_4_ hybrid materials with synergistic visible-light photocatalytic activity, and the uniform AgBr nanoparticles were well dispersed on the g-C_3_N_4_ nanosheets which enhanced the optical activity. What is more, many hybrid materials like g-C_3_N_4_/Ag_2_CO_3_ [138], g-C_3_N_4_/ZnWO_4_ [139], g-C_3_N_4_/Ag_2_O [140], and g-C_3_N_4_/Ag [141] were explored to enhance photocatalytic activity. Sun group obtained mesoporous carbon/g-C_3_N_4_ with remarkably enhanced photocatalytic activity due to enhanced visible light and dye adsorption [142]. Mesoporous g-C_3_N_4_ has been fabricated in the presence of SBA-15 with enhanced photocatalytic activity for methyl orange degradation by Gao et al. [144].

Crystalline carbon nitride nanosheets have been prepared by Lotsch et al. [76] to enhance visible-light hydrogen evolution. The authors stated that morphology and surface area are the two crucial factors governing the photocatalytic performances. An efficient deposition method of growing g-C_3_N_4_ on different electrodes has been developed by Shalom et al. [26]. The successful deposition technique enables the fabrication of many electronic devices based on g-C_3_N_4_. Wu et al. studied the effect of defects in g-C_3_N_4_ on hydrogen evolution and photovoltage. Controlling different types of defects is the key to improve the catalytic performance [27].

He et al. [20] utilized polydopamine/g-C_3_N_4_ composites to produce hydrogen from water with superior activity. DFT calculations were used to obtain the band structure of g-C_3_N_4_ with nonmetal element doping, including boron, oxygen, phosphorous, and others. Phosphorus was predicted to be suitable as a doping element in g-C_3_N_4_ because it can decrease the bandgap of g-C_3_N_4_ from 2.7 to 2.31 eV without any mid-gap states [28].

Shiraishi et al. [35] reported a g-C_3_N_4_ photocatalyst to reduce O_2_ to H_2_O_2_ via a two-electron route under visible light. Fang et al. [49] demonstrated that the nitrogen self-doped g-C_3_N_4_ can significantly enhance the catalytic activity (1.8 times) of hydrogen evolution and modify the optical and electronic properties with respect to the un-doped g-C_3_N_4_. Sun’s group reported ultrathin g-C_3_N_4_ nanosheets/graphene nanocomposites as a highly efficient electrocatalyst for oxygen evolution reaction. They revealed that the high oxygen evolution reaction activities resulted from pyridinic-N-related active sites [121]. They also developed 3D porous supramolecular architecture based on g-C_3_N_4_ nanosheets/graphene oxide as a highly efficient electrocatalyst for oxygen reduction reaction [122].

Besides metal-free materials such as graphene or carbon dots, various metal oxides and sulfides have been coupled with g-C_3_N_4_ for enhancing photocatalytic performances. For example, Chen et al. demonstrated that the g-C_3_N_4_/Ag/TiO_2_ heterostructure microspheres were successfully achieved with enhanced photocatalysis performances [36]. Gu et al. [100] obtained the g-C_3_N_4_/TiO_2_ nanosheets with high reactive (001) facets by a hydrothermal method, accompanied by a remarkable enhancement of photocatalytic capability in degradation of organic molecules under visible and UV light. Wang et al. successfully fabricated g-C_3_N_4_/BiPO_4_ g-C_3_N_4_ photocatalyst. The hybrid structure has been proved by Dong et al. to be a novel photocatalyst for the application of NO purification via an in situ deposition method [37]. Wang et al. explored the enhanced photocatalytic mechanism for the hybrid g-C_3_N_4_/MoS_2_ nanocomposites by DFT calculations. DFT calculations show these hybrid catalytic nanocomposites indeed have a higher absorption (used in the treatment of methylene orange (MO) [101]), with a decent photocatalytic performance and better separation of photo-generated carriers [38]. Ye’s group has demonstrated a zirconium-based metal–organic framework (Uio-66)-g-C_3_N_4_ nanosheet compound via a facile self-assembly method. Photocatalytic CO_2_ reduction activities were greatly enhanced. The photo-generated electron can be transferred from the g-C_3_N_4_ nanosheets to Uio-66 for better reduction of CO_2_ [48].

**Scheme 1:**
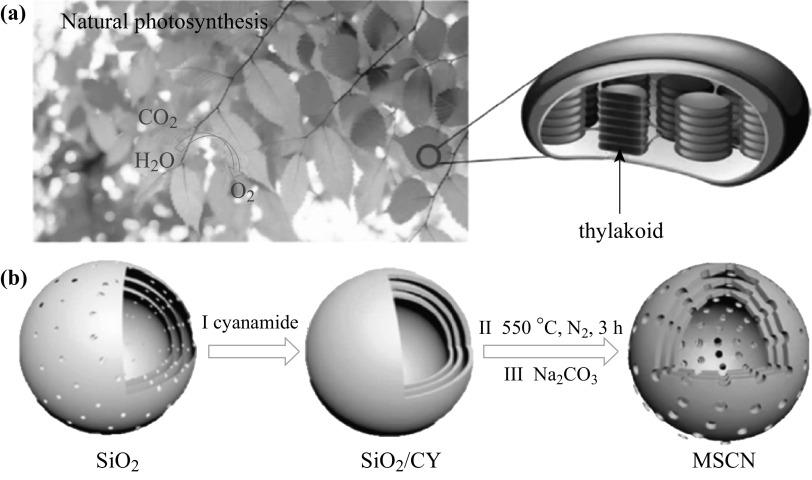
**a** Natural photosystem with green leafs, and the enlarged figure (*right*) depicts the light conversion in the stacked thylakoids. **b** Schematic illustration for the preparation of MSCN nanocapsules. Adapted with permission from Ref. [14]. Copyright 2017 American Chemical Society

Ye’s group was inspired by the natural photosynthesis process and explored an environmental “phosphorylation” strategy to improve the photocatalytic performance [48]. A hydrogen generation rate of 947 µmol h^−1^ and a quantum yield of 26.1% at 425 nm were achieved. This is the highest record for g-C_3_N_4_-based photocatalyst [53]. Recently, Tong et al. were inspired by the function of thylakoids in a natural photosynthesis system as shown in Scheme 1a. They successfully fabricated a multishell g-C_3_N_4_ nanocapsule photocatalysts with enhanced light harvesting and electron transfer properties. The overall synthesis procedure is illustrated in Scheme 1b. The triple-shell g-C_3_N_4_ can produce hydrogen as much as 630 µmol h^−1^. This success potentially produces a new generation of solar devices for hydrogen production.

Molecular doping not only plays an indispensable role in regulating the bandgap and electron structure of g-C_3_N_4_, but also controls the physical and chemical properties of g-C_3_N_4_. The bottom-up copolymerization method allows a large selection of structurally matched organic anchoring groups to be integrated into the g-C_3_N_4_ tri-triazine backbone to design highly efficient photocatalysts with the desired chemical composition and bandgap. It is expected that the modified g-C_3_N_4_ nanocrystals will provide an insightful view of the sustainable use of solar energy in chemistry because of the interesting new features that provide a new avenue for the study of heterogeneous catalysis.

g-C_3_N_4_ has a moderate bandgap of 2.7 eV, which makes it active in the visible region. Thermodynamic losses and overpotentials are usually considered in the photocatalytic process, the bandgap of 2.7 eV lying in between 2 eV (water splitting with enough endothermic driving forces) and 3.1 eV (visible-light absorption). What is more, g-C_3_N_4_ has a suitable conduction band position for various reduction reactions. The CB of g-C_3_N_4_ is more negative than H_2_-evolution, CO_2_-reduction, and O_2_-reduction reactions, revealing that the photo-generated electrons in g-C_3_N_4_ possess a large thermodynamic driving force to reduce small molecules like H_2_O, CO_2_, and O_2_. Therefore, the suitable electronic band structures of g-C_3_N_4_ are favorable for its applications as catalyst, such as water splitting, CO_2_ reduction, oxygen reduction reaction, oxygen evolution reaction, pollutant degradation, and organic synthesis.

*Advantages and disadvantages* Bandgap is crucial for photocatalytic applications. g-C_3_N_4_ has a direct moderate bandgap of 2.7 eV which has visible-light activity besides its advantages of low cost, metal-free, 2D layered structure, easy fabrication, and high stability. Unfortunately, the bulk g-C_3_N_4_ generally exhibits the low photocatalytic efficiency due to some serious drawbacks of g-C_3_N_4_ material, such as the high electron–hole recombination rate, low surface area, insufficient visible absorption, few active sites for interfacial reactions and low charge. Among various design strategies, heterojunction construction (especially for Z-scheme construction) and porosity design (microporous, mesoporous, and macroporous) have been employed. Based on present g-C_3_N_4_ materials, photocatalytic CO_2_ reduction seems to be more promising in developing practical g-C_3_N_4_-based photocatalysts.

### g-C_3_N_4_ Sensing

It is well known that the polymeric g-C_3_N_4_ exhibits photoluminescence (PL) properties similar to many semiconductors materials. g-C_3_N_4_ emits a blue PL around 450 nm when dissolved in solvents under UV light irradiation due to its direct bandgap of 2.7 eV, which can be explained as the transition of the s-triazine ring [39]. Luminescent g-C_3_N_4_ (nanosheets and nanodots) can simply be regarded as nitrogen-rich carbon dots, although the quantum yield of g-C_3_N_4_ (up to 40%) is lower than carbon dots (up to 90%) [102]. g-C_3_N_4_ nanostructures exhibit a higher stability than the carbon dots, therefore potentially providing more practical applications. However, we are still far from the success of improving the quantum yield and understanding the precise PL mechanism of g-C_3_N_4_.

**Scheme 2:**
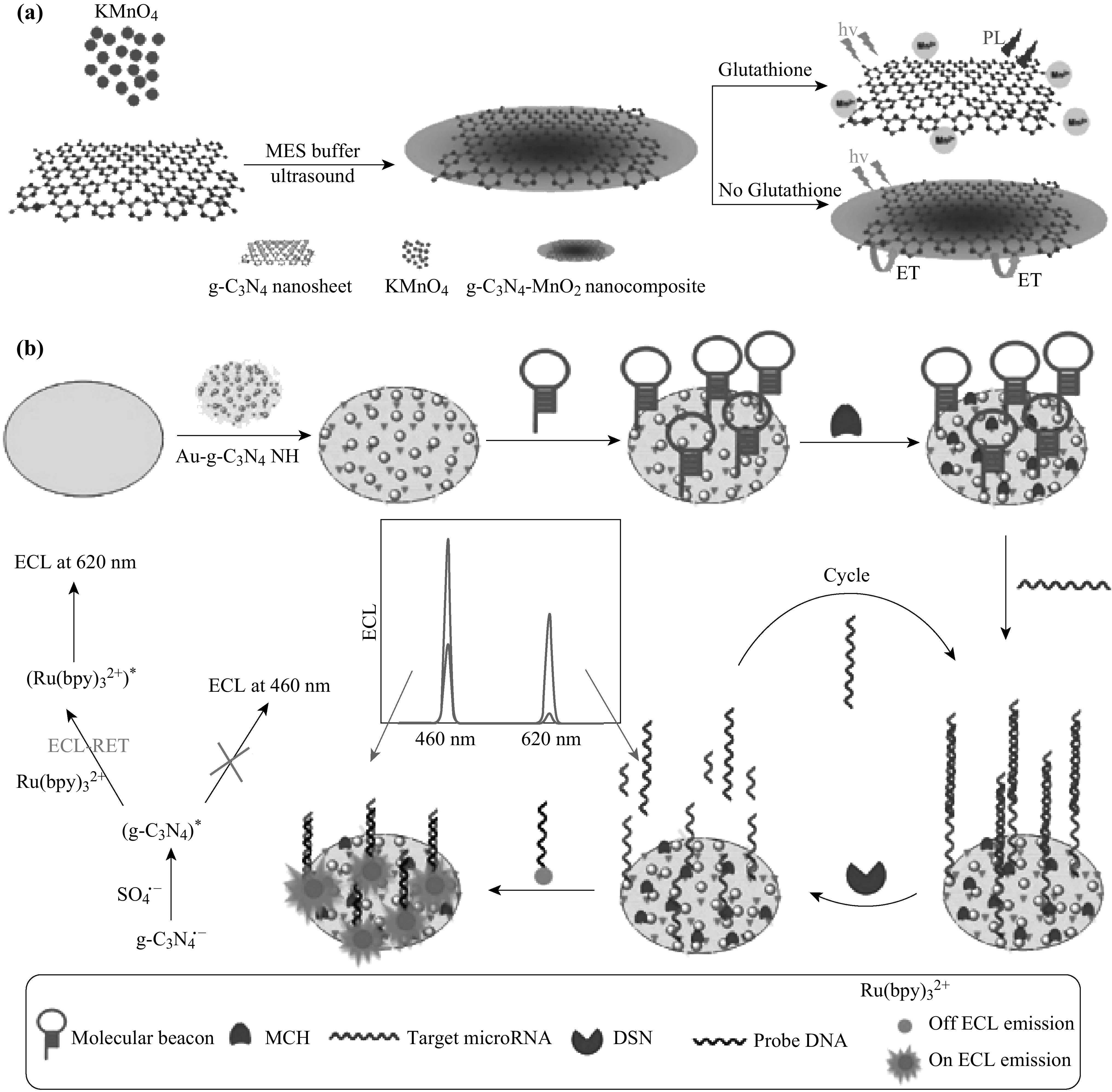
**a** Schematic representation of g-C_3_N_4_/MnO_2_ nanocomposite for sensing of GSH. Reproduced with permission from Ref. [107]. Copyright 2014 American Chemical Society. **b** Schematic illustration of the dual-wavelength ratiometric ECL-RET biosensor configuration strategy. Reproduced with permission from Ref. [114]. Copyright 2016 American Chemical Society

Based on the unique PL property of g-C_3_N_4_, g-C_3_N_4_ nanosheets have a strong response to copper ions [74] as turn-off chemical sensors. Since the chemical reduction of Cu^2+^ to Cu^+^ lies between the conduction band and valence band of g-C_3_N_4_, the PL of g-C_3_N_4_ can be quenched with a low detection limit of 0.5 nM [103]. A similar mechanism has been explained in previous work [104]. In that work, a relatively low detection limit of 0.04 nM has been achieved by CdTe QDs. Besides copper ions, PL of g-C_3_N_4_ can also be quenched by other metal ions like Fe^3+^, Ag^+^, Hg^2+^, and Cr^2+^ [89, 90, 105–110]. Huang et al. reported the fabrication of g-C_3_N_4_ nanosheets for the selective detection to Cu^2+^ and Ag^+^. Zhang et al. successfully prepared the g-C_3_N_4_ QDs as effective fluorescent probes for the detection of Fe^3+^ and Cu^2+^. Cao et al. developed the g-C_3_N_4_ nanodots via a microwave-assisted approach. The produced nanodots were utilized as turn-off sensors for mercury ions with a detection limit of 0.14 μM [89]. Shao’s group successfully synthesized the oxygen- and sulfur-co-doped g-C_3_N_4_ nanodots via a hydrothermal method, which has a lower detection limit of mercury ions (0.37 nM) [90]. Sun’s group successfully fabricated Fe-doped g-C_3_N_4_ nanosheets with a highly sensitive optical detection of glucose due to the chelation of Fe^3+^ with N [123]. Moreover, they demonstrated the ultrathin g-C_3_N_4_ nanosheets can be utilized as electrochemical glucose biosensing with a detection limit of 11 μM [124]. Rong et al. explored turn-off–turn-on sensors using g-C_3_N_4_ nanosheets, Cr and ascorbic acid. After Cr quenches the PL signal of g-C_3_N_4_, the addition of ascorbic acid can recover the PL signal due to the oxidation–reduction between Cr and ascorbic acid. Yang’s group fabricated a g-C_3_N_4_ nanosheet/MnO_2_ sandwich nanocomposite via a one-step approach. The fabricated composites could turn-on the fluorescent probes of glutathione (GSH) with a high selectivity due to fluorescence resonance energy transfer (FRET), as shown in Scheme 2a [111]. GSH is possibly a suitable recovering agent of the PL signal of g-C_3_N_4_ as well. Xu et al. reported a g-C_3_N_4_/Hg system without PL signal, which can be switched on by an introduction of GSH. The system may be employed for detection of GSH in various food samples [112]. Based on FRET, g-C_3_N_4_ was found to detect riboflavin (RF) because g-C_3_N_4_ acts as a donor of energy transfers and RF as an acceptor [113]. A turn-on g-C_3_N_4_-based long-persistent luminescent probe for detection of biothiols was reported by Tang et al. [115] for the first time. This long-persistent luminescence allows the detection without external illumination. Qiao’s group has successfully prepared the proton-functionalized ultrathin g-C_3_N_4_ nanosheets which can interact with heparin, therefore achieving the lowest detection limit of 18 ng mL^−1^ [52].

Feng et al. successfully fabricated Au-nanoparticle-functionalized g-C_3_N_4_ nanosheets coupled with Ru(bpy)_3_^2+^. The coupled g-C_3_N_4_ nanosheets can be employed for RNA detection based on dual-wavelength electrochemiluminescence (ECL), as shown in Scheme 2b. Au/g-C_3_N_4_ composites exhibit an emission at 460 nm and g-C_3_N_4_/Ru(bpy)_3_^2+^ at 620 nm. Here, g-C_3_N_4_ acts as a donor of energy transfer and Ru(bpy)_3_^2+^ as an acceptor for highly sensitive and selective detection of target miRNA (ECL signals quenching at 460 nm and increasing at 620 nm) [114].

In addition to detecting metal ions and bio-molecules by g-C_3_N_4_, g-C_3_N_4_ can also respond to temperature [116]. Debanjan et al. reported a temperature sensor based on the PL of g-C_3_N_4_. They found that as the temperature increased, the intensity of PL decreased.

### g-C_3_N_4_ Imaging

Non-toxicity, metal-free, high stability, and high PL quantum yield enable g-C_3_N_4_ nanosheets and nanodots to be promising candidates for cell imaging. Xie’s group demonstrated the preparation of ultrathin g-C_3_N_4_ nanosheets for bio-imaging applications [65]. They found that g-C_3_N_4_ nanosheets have no significant effects on the HeLa cell viability even at a high concentration. The same group further developed the single-layered g-C_3_N_4_ QDs for both one-photon and two-photon cell imaging as long as the QDs can pass through the nuclear pore and enter into the nuclei [87]. Singlet oxygen, being one of the most important reactive oxygen species (ROS), could be generated in the presence of g-C_3_N_4_ as a photosensitizer [95].

Recently, Yang’s group reported the NIR-driven g-C_3_N_4_/up-conversion nanoparticle (UCNP) composite for efficient bio-imaging and photodynamic therapy (PDT) [117]. In this application, g-C_3_N_4_ functions as a sensitizer to absorb the UV light converted by UCNPs from 980 nm NIR light. The generated ROS causes the tumor to shrink or disappear effectively without any side effects from the irradiation [117]. However, long-time irradiation of 980 nm NIR light can cause overheating of tissues; therefore, an 808 nm laser light is more suitable for the PDT.

**Fig. 9:**
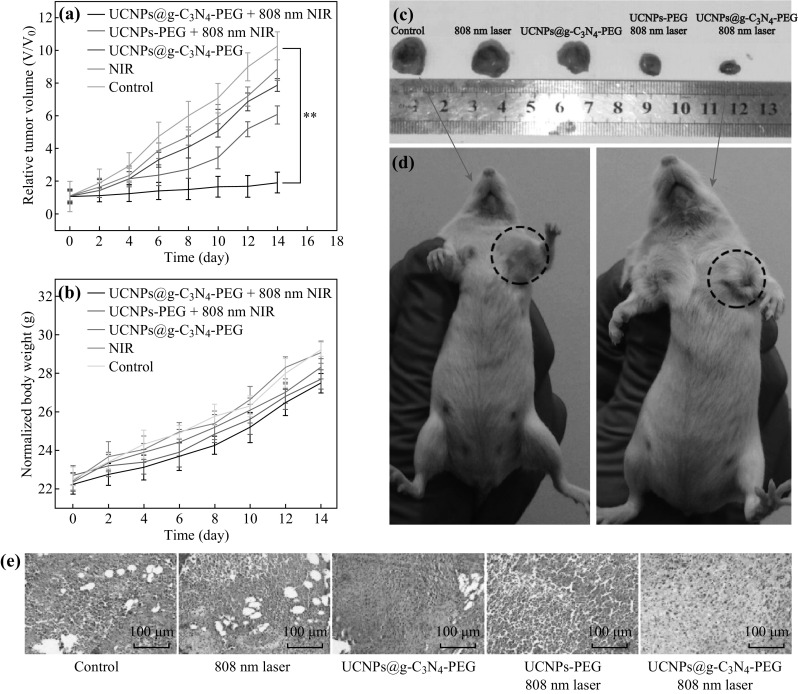
**a** In vivo tumor volume growth curves of mice in different groups after various treatments. **b** Body weight changes of Balb/c mice versus treated time under different conditions. **c** Photographs of excised tumors from representative Balb/c mice after 14 day treatment and **d** the corresponding digital photographs of mice in the control group and “UCNPs@g-C3N4 − PEG with 808 nm laser” group after 14 day treatment. **e** H&E stained tumor sections after 14 day treatment from different groups. Reproduced with permission from Ref. [118] Copyright 2016 American Chemical Society

Feng et al. [118] fabricated a novel core–shell structure (UCNP/g-C_3_N_4_) for phototherapy and imaging applications. Mesoporous g-C_3_N_4_ was coated outside of the shell of UCNPs, generating a large amount of ROS due to the large surface area of g-C_3_N_4_ under the 808 nm laser irradiation. *In vivo* experiments were conducted as shown in Fig. 9. The factors affecting the efficiency of sensing and imaging are mainly the quantum yield, functionalization, PL and optical properties, stability, and toxicity.

### g-C_3_N_4_-Based LED

**Fig. 10:**
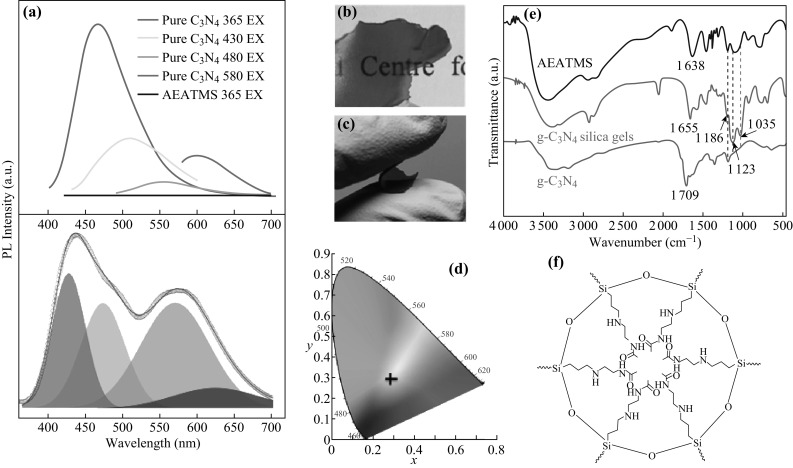
**a** Photoluminescence spectrum of the g-C_3_N_4_/silica gels excited at 365 nm displaying four peaks (430, 480, 580, and 627 nm) in the visible regime. **b**, **c** Photographs of a free-standing g-C_3_N_4_/silica-gel membrane, displaying both good transparency **b** and flexibility **c**. **d** CIE-1931 chromaticity diagram showing the emission from the typical g-C_3_N_4_/silica gels (marked by the *black cross*) excited at 365 nm. **e** FTIR spectra of AEATMS, g-C_3_N_4_-silica gels, and g-C_3_N_4_ particles. **f** Schematic drawing of an AEATMS-capped g-C_3_N_4_ particles in the g-C_3_N_4_/silica gels. Reproduced with permission from Ref. [119]. Copyright 2016 Wiley

Although the PL properties of g-C_3_N_4_ have been investigated in the past fifteen years, the solid-state lighting of g-C_3_N_4_-based materials is still at an infancy stage. Various investigations about the g-C_3_N_4_-based solid-state lighting such as white-light-emitting diodes (WLEDs) have been carried out. Wang et al. fabricated the g-C_3_N_4_/silica gels for WLEDs application (Fig. 10a–d). In this work, using a one-step heat treatment approach confirmed by the FTIR, g-C_3_N_4_ was found to be covalently bonded with silica gels (Fig. 10e, f). The g-C_3_N_4_/silica gels obtained possess emerging properties with respect to bare g-C_3_N_4_, including water-resistance, high transparency, high flexibility, and white light emission under UV irradiation. The mechanism for white light emission can be ascribed to surface passivation by silica [119]. Bayan et al. [120] prepared a g-C_3_N_4_ sheet/ZnO nanorod hybrid for WLEDs by combining the emissions of g-C_3_N_4_ and ZnO nanorods to achieve a broad emission. Last but not the least, Gan et al. studied the origins of broad PL of g-C_3_N_4_ for the WLEDs applications. They demonstrated that the broad PL from g-C_3_N_4_ is attributed to band-to-band transitions in the tri-s-triazine rings. This novel work can help the understanding of the PL mechanism and accelerate the progress of WLEDs-based g-C_3_N_4_.

## Conclusion and Outlook

This review summarizes the recent advances of g-C_3_N_4_-based structures and applications including catalyst, chemical and biosensing, imaging, and LEDs. The performances of g-C_3_N_4_ are mainly based on their surface state (defects, function groups, and doping) and structures (porosity, thickness, and morphology). Although a significant advancement has been made for the development of highly efficient g-C_3_N_4_-based photocatalysts, there are still considerable problems that require further investigations, including the catalytic rate and design.

2D polymeric g-C_3_N_4_ materials featuring low cost, metal-free, environmental friendly, moderate bandgap, high chemistry activity, and high stability have only been studied for the past few years (from fundamental research to practical applications). We believe that more emerging properties and applications of g-C_3_N_4_ are around the corner. Integrations between experimental research and theoretical approaches will advance the research progress of g-C_3_N_4_ to a large extent. As the exploration of g-C_3_N_4_ that is still in its infancy, there are several remaining key challenges that must be met in near future, including porous nanostructures for the drug loading and delivery, improvement of electronic conductivity, memory device fabrication, solid-state lighting, energy conversion, and wearable sensors.
